# Serum Metallothioneins in Childhood Tumours—A Potential Prognostic Marker

**DOI:** 10.3390/ijms140612170

**Published:** 2013-06-06

**Authors:** Jarmila Kruseova, David Hynek, Vojtech Adam, Rene Kizek, Richard Prusa, Jan Hrabeta, Tomas Eckschlager

**Affiliations:** 1 Department of Paediatric Haematology and Oncology, 2nd Medical Faculty and University Hospital Motol, V Uvalu 84, Prague CZ 150 06, Czech Republic; E-Mails: jarmila.kruseova@fnmotol.cz (J.K.); janhrabeta@gmail.com (J.H.); 2 Department of Chemistry and Biochemistry, Faculty of Agronomy, Mendel University in Brno, Zemedelska 1, Brno CZ 613 00, Czech Republic; E-Mails: d.hynek@email.cz (D.H.); vojtech.adam@mendelu.cz (V.A.); kizek@sci.muni.cz (R.K.); 3 Central European Institute of Technology, Brno University of Technology, Technicka 3058/10, Brno CZ 616 00, Czech Republic; 4 Department of Medical Chemistry and Clinical Biochemistry, 2nd Medical Faculty and University Hospital Motol, V Uvalu 84, Prague CZ 150 06, Czech Republic; E-Mail: richard.prusa@fnmotol.cz

**Keywords:** serum metallothioneins, children, solid tumours, treatment, differential pulse voltammetry Brdicka reaction

## Abstract

Metallothioneins (MT) are low molecular weight, cysteine-rich proteins maintaining metal ions homeostasis. They play a role in carcinogenesis and may also cause chemoresistance. The aim of the study was to explore the importance of MT serum levels in children suffering from malignant tumours. This prospective study involves examination of 865 samples from 172 patients with malignant tumours treated from 2008 to 2011 at University Hospital Motol. MT serum levels were determined using differential pulse voltammetry–Brdicka reaction. Mean MT level was 2.7 ± 0.5 μM. There was no statistically significant difference between MT levels in different tumours. We also did not find any correlation between MT levels and response to therapy or clinical stages. However, we found a positive correlation between MT levels and age (*p =* 0.009) and a negative correlation with absolute lymphocyte number (*p =* 0.001). The fact that patients who had early disease recurrence had lower MT levels during the treatment (complete remission 2.67 *vs.* recurring 2.34, *p =* 0.001) seems to be important for clinical practice. Accordingly we believe that there is benefit in further studies of serum MT levels in tumours.

## 1. Introduction

Metallothioneins (MTs) are low molecular weight cysteine rich proteins, which have naturally occurring Zn^2+^ in both binding sites [1]. They are found in the cytoplasm and various subcellular organelles, particularly in liver, kidney and intestinal cells [2,3]. Four major metallothionein isoforms have been identified in mammals (MT 1–4). MT-1 and 2 are ubiquitous, and their main functions are the regulation of copper and zinc metabolism and the detoxification of heavy metals. They perform several functions of gastrointestinal tract, are involved in cell transcription, and play a role in immune function. MT-3 (also called neuronal growth-inhibitory factor) is found in the brain and small levels are present in the pancreas and intestines. MT-3 is synthesized primarily in the astrocytes of cortex, brainstem and spinal cord [4]. Its main function is a growth inhibitory factor in brain [5]. MT-4 is located in the epithelial cells of skin, tongue and stomach. It participates in the regulation of stomach acid pH, taste and texture discrimination of the tongue, and helps to protect the skin against damage from UV light [6,7]. Experimental study has shown reduced neuronal protection against oxidative stress-induced damages in MT knockout mice [8]. However, mice with deletions of both the *MT-1* and *MT-2* genes do not exhibit an altered phenotype under normal conditions [9–12]. MT-1 and MT-2, unlike MT-3 and MT-4, are highly inducible in mammalian cells by different stimuli (heavy metals, cytokines). A significant association between metal levels, MT expression, and disease was found in various tissues [6,7].

There are some experimental and clinical precedents for suggesting that MTs can be exported and taken up by cells through a receptor-mediated mechanism [13]. High levels of MTs were detected in secreted pancreatic juice after pilocarpine stimulation in mice [10]. Biliary excretion of MT-1 rose in rats fed with a high copper diet [12]. MTs were released after the induction of differentiation of fibroblastic preadipocytes into adipocytes *in vitro*) [11]. MTs were detected in human seminal plasma secreted predominantly from the prostate [14].

MTs could be very important for patients who are undergoing anti-cancer therapy. They have antiapoptotic, antioxidant, proliferative and proangiogenic effects that are important in oncogenesis, tumour progression and response to anticancer therapy [1,3,15–18]. MTs are also involved in resistance to cytostatic drugs, because they reduce drug uptake, increase drug efflux and participate in DNA repair [17–22]. There is a great deal of information about cellular MT content in different cancers and about its prognostic significance [1,23]. However, there is limited knowledge about serum levels in cancer patients and so far, there are no conclusive data on the significance of MT serum level in child patients. MT-2A is the most expressed MT human isoform because of the binding ability of enhancers in the *MT-2A* promoter region [24]. However, the information on serum levels of individual isoforms is still lacking.

The aim of this study is to explore relationships between MT serum levels and type of tumour, stages and response to therapy in children suffering from solid tumours. We also were looking for correlation between MT levels and other laboratory findings.

## 2. Results

### 2.1. MT in Serum

**Figure 1:**
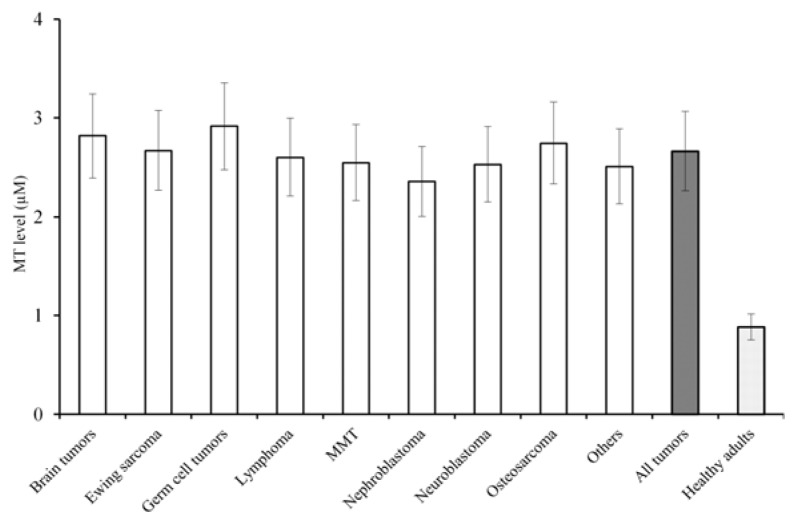
MT level/mean ± s.d./in different childhood malignant tumours. Hatched column: all tumours; doted columns: healthy adult volunteers. There were no statistically significant differences between different tumours, difference between MT levels in all malignant tumours and in healthy volunteers was significant. MMT: Soft tissue sarcoma (Malignant mesenchymal tumours).

The mean MT level in children with malignant tumours was 2.67 ± 0.5 μM. There were no statistically significant differences between different tumours in our group of patients (Figure 1). The highest mean MT levels were found in germ cell tumours (MTs 2.94 ± 0.9 μM) and the second highest levels were determined in brain tumours (2.82 ± 0.5 μM) but the differences are not statistically significant and are probably caused by age distribution. We previously published average MT levels in serum of healthy adult volunteers detected also using the Brdicka method. In the sample (mean age 27 years), MT level was 0.52 ± 0.2 μM [25]. Milnerowicz and Bizoń found similar results in healthy adult volunteers using enzyme-linked immunosorbent assay [26] and Singh and Hanson found lower MT levels in 50 normal children (aged 2–11 years) by immunoassay (approx. 2 mg/L) [27]. Somewhat higher were MT levels detected by differential pulse voltammetry–Brdicka reaction found in a group of children (age 3–17 years) with various renal diseases with normal creatinine levels (MTs 1.42 ± 0.09 μM) [28]. Those levels in healthy adult controls are approximately five times lower than the average (2.67 ± 0.5 μM) MT levels found in our group of patients. The MT levels in the group of children with renal diseases were roughly half that of values in children suffering from malignant tumours. Both those differences were statistically significant (*T*-test, *p <* 0.01). The MT levels found in a group of children with nonmalignant kidney diseases were approximately half of levels in our group with malignant tumours. We found highly significant positive correlation between MT levels and age that was independent of tumour type. Figure 1 depicts a general linear model with dependent variable MT level, between subjects factor type of tumour and covariate age, displaying significance of covariate effect age *p =* 0.009 (*F* = 7.05, df = 1), parameter estimate of age B = 0.023 (positive).

### 2.2. MT Relationship to Course of the Disease

**Figure 2:**
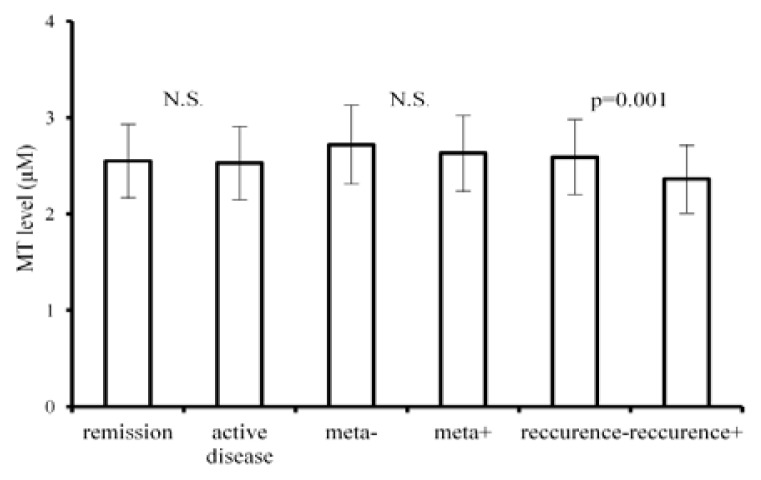
Relation of MT level/mean ± s.d./to clinical parameters. N.S.: not significant. *p =* 0.001: statistically significant on the level of 0.1%. There were no statistically significant differences between MT levels in patients with active disease and in remission or in patients with generalized disease and with cancers without metastasis.

**Figure 3:**
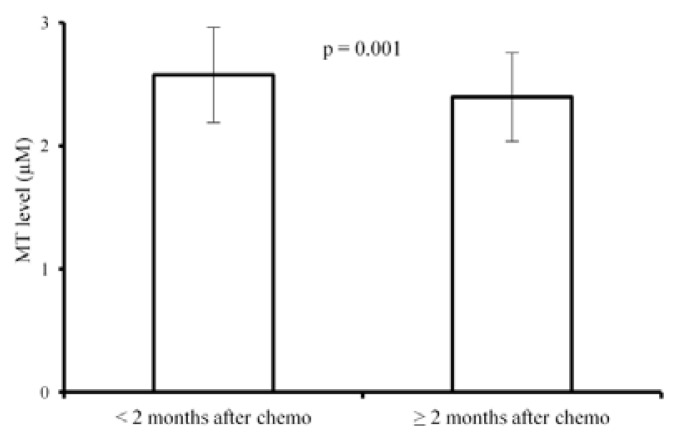
MT levels/mean ± s.d./in group of patients <2 months after finishing chemotherapy compared to patients ≥2 months after finishing chemotherapy [paired *T*-test (*t* = 3.53, df = 75) *p =* 0.001].

We did not find any differences in MT levels among patients with tumours before starting therapy, with tumour and/or metastases during therapy or with progressive disease (group of “patients with active disease”) and patients in complete remission (group without “active disease”). There were no statistically significant differences in MT levels in patients with metastatic diseases in comparison with levels in patients with localized cancer. The majority patients displaying lower MTs levels had early recurrence of disease. Average MT levels during all follow up period before detection of recurrence was 2.34 μM, *versus* complete remission 2.67 μM (*p* = 0.001). These results are shown in Figure 2. We, however, found significant decrease of MT levels in children during follow up ≥2 months after the end of the treatment as compared with levels at the end of therapy (*p =* 0.001, Figure 3).

### 2.3. MT Levels and Chemotherapy

**Figure 4:**
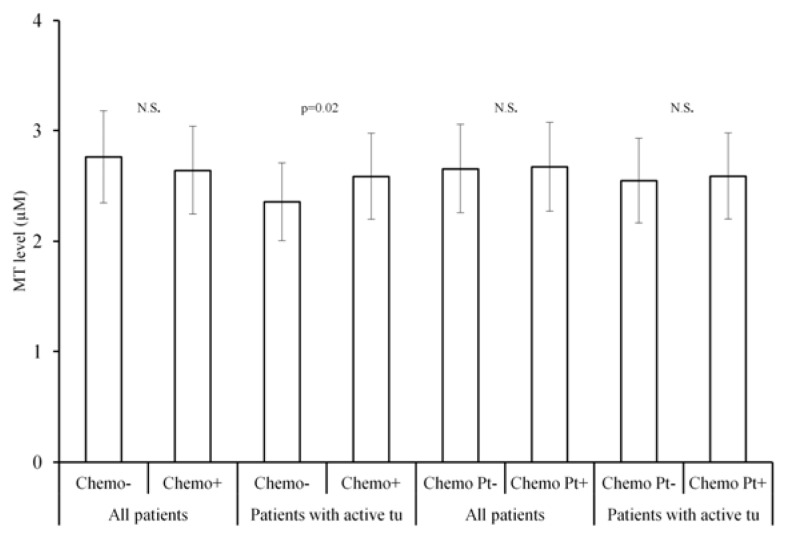
MT levels in relation to chemotherapy. N.S.: not significant. *p =* 0.02: statistically significant on the level of 2%. The only significant difference was found between levels in patients with active tumour (before therapy, partial remission, progressive disease) with and without chemotherapy. There was no relationship to chemotherapy with platinum cytostatics.

MT levels in patients with active disease were higher in those treated with chemotherapy than without (*p =* 0.02). There was no significant difference in MT levels in patients treated by protocols containing platinum cytostatics and treated without those cytostatics (Figure 4).

### 2.4. Correlation of MT Levels with Other Biochemical Parameters

There was a negative correlation between MT levels and absolute lymphocyte count (*p =* 0.001) and positive correlation between MT and creatinine levels (*p =* 0.003). Correlation between haemoglobin and MT levels was below the level of statistical significance (*p =* 0.085). Patients with CRP above normal levels exhibited no difference in MT level compared to those with normal CRP (*p =* 0.912). Summary of the results obtained is shown in Table 1. Correlations of MT levels with other laboratory findings mentioned in Materials and methods were not significant.

## 3. Discussion

Numerous studies have been carried out to understand the relationship between MT in cells and cancer. Most of these have focused on adults [6,18,29–33] and far fewer on children [34–37]. Studies in adults have shown that cellular MT could serve as prognostic markers in some tumours [17,38–43]. Relationships have been described between MT and p53 status [1], increased tumour grade [44,45] and metastases development [1,46]. However what has been proved in adults is not so clear in childhood cancers. There is one study in children with osteosarcoma, where the authors found differential expression of MT in biopsies [36] and they suggested that MT might play an important role in development of disease. Another study on children suffering from leukaemia showed that patients with acute lymphoblastic leukaemia whose blasts express MT tended to have shorter disease-free survival compared with the MT negative ones [37]. A further report on patients with neuroblastoma demonstrated only limited value of MT for prediction of therapeutic response [34].

MT levels in serum have seldom been researched in cancer patients. They have been used for monitoring intoxication by metals (lead, cadmium) [47]. Previously, our group has published three publications on MT in serum in adult cancer. Increased MT serum levels in prostate cancer patients were found to be negatively correlated with their Gleason score [48]. MT levels in patients suffering from head and neck spinocellular cancer demonstrated correlation with tumour grade and clinical stage [49,50]. In another study hepatocellular carcinoma serum MT levels were decreased compared to the control group [51]. To our knowledge no work has yet been published on MT serum levels in child cancer patients.

Our study covered a wide range of child cancers. MTs were found not to act as a prognostic marker of treatment response in any of these diseases. Patients with metastases or progressive disease did not exhibit different MT levels either. Differences between different types of tumours were not significant. The highest mean MT levels we found were in germ cell tumours (MT 2.94 ± 0.9 μmol·dm^−3^) most likely because these included a higher proportion of older patients. The second highest were in brain tumours (2.82 ± 0.5 μmol·dm^−3^)—probably because the brain, in general, contains higher amounts of MT [16,52,53], but age distribution may also play a role. During anticancer therapy there are many different pathological reactions (tissue damage induced by cytostatics and/or radiotherapy, supportive therapy including antibiotics and antimycotics, erythropoetin and/or filgrastim, infections, tumour behaviour, *etc.*). Each of these contributes to various and hence continuously varying levels of oxidative stress. MTs are known to function as one of the main antioxidant defence systems [1,54,55]. Changes of MT levels caused by therapy and/or its complications mentioned above are probably the main reason why correlation between tumour activity and MT levels is not significant. We have not found significant correlation between tumour activity and MT levels.

The finding that low MT levels during treatment were closely correlated with early relapses may be important for clinical practice. Peyere *et al.* found that up to 80% of children with recurrence of ependymoma have down-regulated *MT-3* genes, not caused by *MT* gene deletion or promoter methylation. *MT-3* expression was restored by histondeacetylase inhibitor or zinc treatment [35]. One may suggest that down-regulation could be a key cause of low MT levels in patients suffering from recurrence, although from twelve recurrent tumours, only three were brain tumours and it is not known if other MT isoforms expression is also down regulated in recurrent tumours of other organs.

The explanation of slow decline (2 months) of MT levels after completing therapy is long recovery reactions in the organisms after the end of treatment. We choose a two month limit because it is an interval in which haematopoiesis and immunity is usually reconstituted after conventional doses of chemotherapy, and one may speculate that other systems are also normalized during this interval [56]. This suggests that chemotherapy influences MT levels by multiple mechanisms e.g. the damage of different organs and systems, the increasing risk of infections, and/or by the production of reactive oxygen species. It will be necessary to observe these patients over longer periods to determine when MT levels return to normal. We suggest that changes of MT levels could be considered as a promising marker for late relapses. In our study we did not have patients who suffer late recurrence more than 2 years after the end of treatment. The fact that MT levels declined during the two months following the end of treatment supports our view that the anticancer and supportive therapy significantly affects the serum levels of MT.

MT plays an important role in chemoresistance and not only to metal containing drugs. Their expression may be increased not only by metals but by other stimuli e.g. glucocorticoids, catecholamines, free radicals, tumour necrosis factor α, interleukins-1, -2 and -6 [1,17]. Production of some those MT-inducing biologically active molecules is stimulated by chemotherapy. This may explain increased MT levels in patients with “active tumours” treated with chemotherapy.

The findings of increased MT levels in patients with active disease (with tumours before starting therapy, with tumour and/or metastases during therapy or with progressive disease) but not in remission treated by cytostatics may be explained by the production of MT by cancer cells stimulated by chemotherapy. MTs are able to bind platinum-based cytostatics and thereby reduce their cytotoxic effect. Chemoresistance to platinum anti-tumour compounds is mediated through several mechanisms. One of them is the transfer of platinum from cisplatin and carboplatin to MT that results in inactivation of those drugs. Cultivation of neuroblastoma cells resistant to cisplatin in medium with cisplatin or carboplatin, has been shown to significantly increase intracellular MT levels [1]. However in a sensitive cell line only insignificant increases in MT were detected after cultivation with the same concentrations of cisplatin or carboplatin [1]. Another study showed that a cisplatin-resistant ovarian cancer cell line exposed to cisplatin manifested a nuclear MT expression [57]. In hepatoblastoma patients treated with carboplatin, it was verified that non-responders had a higher percentage of MT-positive tumour cells [58]. The significance of MT expression to resistance of gastric cancer to cisplatin was verified by Suganuma *et al.* [59]. MT upregulation was detected in medulloblastoma and rhabdomyosarcoma cells with induced resistance to the alkylating drug BCNU [60]. Esophageal carcinomas which do not express MT, respond well to chemoradiotherapy (5-fluorouracil and cisplatin) while cancers with high MT expression are resistant [61]. Women with breast carcinoma treated with chemotherapy (cyclophosphamide, methotrexate, 5 fluorouracil or doxorubicin) had significantly longer survival if their tumours had lower MT expression [42]. Hishikawa and co-workers found that MT negative patients with oesophageal cancer treated with cisplatin experienced increased survival compared to those with MT positive tumours [62].

It has been found that MT expression initially increases with age, but then decreases in people over seventy [63] and the inducibility of MT increase with age in infant rat model was described by Bauerly [64]. Correlation of serum MT levels found in our study may also indirectly confirm this phenomenon. Natale *et al.* showed in animal experiments that low MT explains enhanced susceptibility to neuronal loss after injury in immature brains [53]. It is common knowledge that chemotherapy is more toxic in very young children compared to older children. Our findings of low MT in infant patients could be one of the possible reasons for more serious side effects of chemotherapy and radiotherapy in infants compared to older children.

The possible explanation of decreased MT in patients with higher creatinine levels may be kidney damage, as they are one of the major producers of MTs. MT-1 and MT-2 are produced particularly in kidney, liver, pancreas and intestine [1]. The positive correlation of MT levels and lymphocyte count in cancer patients do not have a clear explanation. Due to significant correlations between serum creatinine and lymphocyte counts and haemoglobin levels, it is difficult to assess whether the relationship between MT and creatinine is primary, or whether there are primary relationships for haematological parameters. It is possible that the induction of MT by cytokines such as interleukin-1, -2 and -6 or tumour necrosis factor, which are produced by lymphocytes, may play a possible role.

It has been found that zinc supplementation influences lymphocyte production [63,65]. The authors looked at changes in large numbers of genes that were involved in zinc homeostasis in peripheral blood leukocytes of children with septic shock. MT expression in children who died was also increased. It was suggested that decline in zinc concentrations among critically ill children was related to shifts in MT expression and low plasma zinc levels were associated with the degree of organ failure [64–66]. Child patients who have low lymphocyte count also have increased risk of sepsis. In our study we noticed that patients with low lymphocyte count had increased MT levels. Three of our patients had life threatening sepsis during leucopoenia and high serum MT levels—2.1 times higher than mean (data not shown), which is in agreement with the studies mentioned above. The fact that CRP did not correlate with MT levels suggests that MTs are not acute-phase proteins, and may be a laboratory marker that is independent of acute-phase.

## 4. Experimental Section

### 4.1. Patients

This prospective study involves examination of 865 samples from 172 patients with malignant tumours treated at the Department for Paediatric Haematology and Oncology, University Hospital Motol from 2008 to 2011. Samples were collected before starting chemotherapy, during chemotherapy and after. Diagnoses and age distribution see Table 2. The group included 71 girls (41%) and 101 boys (59%). Other clinical parameters were: metastatic disease 93 (54.1%), death during follow up 32 (18.6%), recurrence during follow up 12 (7%). The minimal follow up was 18 months and median follow up was 39 m (Table 2).

In our study we investigated the correlation between MT levels and clinical parameters (age, diagnosis, clinical stage, recurrence and response to anticancer therapy). We also compared MT levels examined before, during and after therapy with other laboratory findings: total blood count and urea, creatinine, uric acid, lactate dehydrogenase, transaminases, bilirubin, ferritin, total protein, and C-reactive protein (CRP) determination. Total blood count was examined at the Department of Clinical Haematology, and other biochemical tests at Department of Clinical Biochemistry and Pathobiochemistry, University Hospital Motol, according to standard protocols.

### 4.2. Determination of Metallothioneins

Samples were prepared by heat treatment using an automated pipetting system epMotion 5075 (Eppendorf, Hamburg, Germany) and kept at 4 °C then transferred to the 96 well plates (Eppendorf, Hamburg, Germany) together with 0.2 M phosphate buffer pH 7. This mixture was kept at 99 °C for 15 min. The last step was cooling down of samples to 4 °C. Heating denatures and removes the high molecular weight proteins from samples [67]. MT quantification was determined by electrochemical detection. Differential pulse voltammetric Brdicka reaction measurements were performed using a 747 VA Stand instrument connected to a 693 VA Processor and 695 Autosampler (Metrohm, Herisau, Switzerland), using a standard cell with three electrodes, a cooled sample holder and measurement cell. Measurements were taken on samples cooled to 4 °C (Julabo F25, Julabo, Seelbach, Germany). A hanging mercury drop electrode (HMDE) with a drop area of 0.4 mm^2^ was the working electrode. An Ag/AgCl/3M KCl electrode was the reference and platinum electrode was auxiliary. For data processing VA Database 2.2 by Metrohm was employed. The analysed samples were deoxygenated prior to measurements by purging with argon (99.999%) saturated with water for 120 s. A Brdicka supporting electrolyte containing 1 mM Co(NH_3_)_6_Cl_3_ and 1M ammonia buffer pH = 9.6 was used. The supporting electrolyte was exchanged after each measurement. The parameters of the measurement were as follows: initial potential of −0.7 V, end potential of −1.75 V, modulation time 0.057 s, time interval 0.2 s, step potential 2 mV, modulation amplitude −250 mV, E_ads_ = 0 V, volume of injected sample: 10 μL, volume of measurement cell 2 mL (5 μL of sample + 1995 μL Brdicka solution) [48]. Electrochemical determination of metallothionein using Brdicka reaction is described in detail in our previously published papers [25,50,68–71]. In a Brdicka solution, MT gives four very well separated signals [72,73]: 1/Cat1 (potential app. −1.25 V); 2/Cat2 (potential app. −1.45 V) signals correspond to the reduction of hydrogen at the mercury electrode (hydrogen is generated from the supporting electrolyte; 3/RS_2_Co (potential app. −1.1 V) signal represents response of MT complex to components of Brdicka’s supporting electrolyte; 4/the signal called Co1, at the potential app. −1.0 V, probably relates to the reduction of the RS_2_Co complex. The first three MT signals of Brdicka reaction (RS_2_Co, Cat1 and Cat2) displayed well developed and separated peaks with decreasing MT concentration. Signal Co1 decreased and shifted to more negative potential with decreasing MT [73].

### 4.3. Statistical Analysis

Data were evaluated as parametrical. Kolmogorov–Smirnov test was used for testing of normal distribution of MT levels and it was not rejected (K–S test, *p* = 0.17). Data were expressed as mean ± standard deviation. Differences *p <* 0.05 were considered as significant. Used tests: for comparison of MT levels between two groups independent *t*-test, in paired data paired samples *t*-test, general linear model with 1 between subjects factor and 1 covariate for evaluation of influence of factor with account of age, for correlation of MT levels with other parameters Pearson correlation coefficient or partial correlation coefficient with controlling variable age (correlation of MT with creatinine, because both are growing with age) and for comparison among groups One way ANOVA with Post hoc tests Scheffe was used. IBM SPSS Statistics (Release 20.0, IBM Corporation, Armonk, NY, USA) was used.

## 5. Conclusions

We found that differential pulse voltammetry with automatic sample preparation is suitable for quantification of MT in serum. This method is fast, relatively inexpensive and enables efficient analysis of large numbers of samples, but it is so far unable distinguish different MT isoforms. In spite of the fact that the Brdicka reaction is not sensitive to the single isoforms, specificity of the sample preparation and isolation is higher than 90% [73–75]. There are increased serum MT levels in children suffering from cancer compared to healthy volunteers described in our previous study and a group of children with kidney diseases and also from groups of healthy persons described in literature and their serum MT levels decline after finishing chemotherapy. We found a correlation between MT levels and age and absolute lymphocyte number in our group of patients. There was no correlation with tumour responses and stages. All patients with early relapses had low MT levels. Hence, one may speculate that lower MT levels could be a promising marker for early relapses. Accordingly we believe that there is benefit in further larger studies of serum MT levels in child cancer patients. Further studies should be extended to the examination of individual MT isoforms since they play different functional roles.

## Figures and Tables

**Table 1:** Correlations between laboratory parameters.

Correlations					

		MT	ANL	CRP	Hgb
MT	Pearson r	1	−0.252 **	0.009	0.132 #
	Sig. (2-tailed)	-	0.001	0.912	0.085
	N	172	171	152	171

ANL	Pearson r	−0.252 **	1	−0.126	−0.040
	Sig.(2-tailed)	0.001	-	0.119	0.599
	N	171	174	154	174

CRP	Pearson r	0.009	−0.126	1	0.039
	Sig. (2-tailed)	0.912	0.119	-	0.635
	N	152	154	154	154

Hgb	Pearson r	0.132	−0.040	0.039	1
	Sig. (2-tailed)	0.085	0.599	0.635	-
	N	171	174	154	174

Crea	Pearson r	0.273	−0.381	0.170	0.445
	Sig. (2-tailed)	0.003	0.000002	0.036	0.000001
	N	170	173	153	173

ANL: absolute lymphocyte number; CRP: C-reactive protein, Hgb: haemoglobin, Crea: creatinine.

** *p* < 0.01,

# near the level of statistical significance.

**Table 2:** Clinical characteristics of patients group.

Diagnosis	No. of pat.	Median age	Minimum age	Maximum age	Recurrence	Metastatic	Chemotherapy/Pt cytostaic **
Neuroblastoma	33	1 y 8 m	1 m	15 y	3	24	30/24
Brain tumours	27	9 y 2 m	8 m	15 y 4 m	3	3 *	27/16
Lymphoma	24	15 y 10 m	9 m	18 y 4 m	2	/	24/0
Ewing sarcoma	20	12 y 7 m	1 y 5 m	18 y 3 m	0	3	20/0
Germ cell tumours	14	16 y 4 m	6 m	19 y 6 m	1	10	14/14
Osteosarcoma	12	13 y 1 m	6 y 5 m	16 y 11 m	1	3	12/12
Soft tissue sarcoma	12	10 y 8 m	1 y 8 m	16 y 6 m	1	1	12/0
Nephroblastoma	7	1 y 9 m	9 m	6 y 10 m	1	3	7/2
Other malignant tumours	23	16 y 4 m	6 m	19 y 6 m	0	9	16/2
All	172	9 y 11 m	1 m	19 y 6 m	12	56	162/70

* spinal metastases,

** no of treated by any cytostatic/no of treated by Pt containing cytostatic (cisplatin and/or carboplatin).
